# Modelling Feedback Excitation, Pacemaker Properties and Sensory Switching of Electrically Coupled Brainstem Neurons Controlling Rhythmic Activity

**DOI:** 10.1371/journal.pcbi.1004702

**Published:** 2016-01-29

**Authors:** Michael J. Hull, Stephen R. Soffe, David J. Willshaw, Alan Roberts

**Affiliations:** 1 Institute for Adaptive and Neural Computation, University of Edinburgh, Edinburgh, United Kingdom; 2 School of Biological Sciences, University of Bristol, Bristol, United Kingdom; Northeastern University, UNITED STATES

## Abstract

What cellular and network properties allow reliable neuronal rhythm generation or firing that can be started and stopped by brief synaptic inputs? We investigate rhythmic activity in an electrically-coupled population of brainstem neurons driving swimming locomotion in young frog tadpoles, and how activity is switched on and off by brief sensory stimulation. We build a computational model of 30 electrically-coupled conditional pacemaker neurons on one side of the tadpole hindbrain and spinal cord. Based on experimental estimates for neuron properties, population sizes, synapse strengths and connections, we show that: long-lasting, mutual, glutamatergic excitation between the neurons allows the network to sustain rhythmic pacemaker firing at swimming frequencies following brief synaptic excitation; activity persists but rhythm breaks down without electrical coupling; NMDA voltage-dependency doubles the range of synaptic feedback strengths generating sustained rhythm. The network can be switched on and off at short latency by brief synaptic excitation and inhibition. We demonstrate that a population of generic Hodgkin-Huxley type neurons coupled by glutamatergic excitatory feedback can generate sustained asynchronous firing switched on and off synaptically. We conclude that networks of neurons with NMDAR mediated feedback excitation can generate self-sustained activity following brief synaptic excitation. The frequency of activity is limited by the kinetics of the neuron membrane channels and can be stopped by brief inhibitory input. Network activity can be rhythmic at lower frequencies if the neurons are electrically coupled. Our key finding is that excitatory synaptic feedback within a population of neurons can produce switchable, stable, sustained firing without synaptic inhibition.

## Author Summary

Rhythmic actions like chewing, scratching and walking need to be switched on and off. Once started, rhythms are generated by networks of neurons in the brain and spinal cord which drive muscles. We use computer models of the swimming network in young frog tadpoles to ask how electrically-coupled brainstem neurons with feedback excitation generate rhythmic activity and how this can be switched on and off by sensory stimuli. Young tadpoles swim for several seconds when touched and stop when they contact a solid object. Swimming rhythms can be generated by minimal populations of ~30 reticulospinal neurons on each side of the brainstem. The sensory pathways providing excitatory start and inhibitory stop signals are known. In our model network, based closely on biological evidence, brief synaptic excitation can activate a stable swimming rhythm sustained by mutual excitation among the reticulospinal neurons; swimming can be stopped by brief inhibition. Model networks of more generic neurons with mutual excitation can produce switchable, sustained non-rhythmic firing. We conclude that mutual excitation, providing positive feedback, can enable stable rhythmic or non-rhythmic firing in small neuron populations that can be rapidly turned on and off by sensory inputs.

## Introduction

Many rhythmic motor patterns are generated within the nervous system by networks of neurons [1–6]. The mechanisms generating activity are well studied across vertebrates and invertebrates and computer network simulations can produce sustained output patterns similar to those in real animals: locust flight [7], salamander walking [8] and swimming in sea slug [9, 10], leech [4, 11], lamprey [12] and tadpole [13]. Despite this, key questions remain: the relative importance of cellular pacemaker properties versus network properties for rhythm generation; the roles of electrical coupling in neuronal synchronisation; whether feedback from mutual excitatory synapses can sustain activity; and finally, how rhythmic activity is controlled by sensory stimuli.

**Fig 1:**
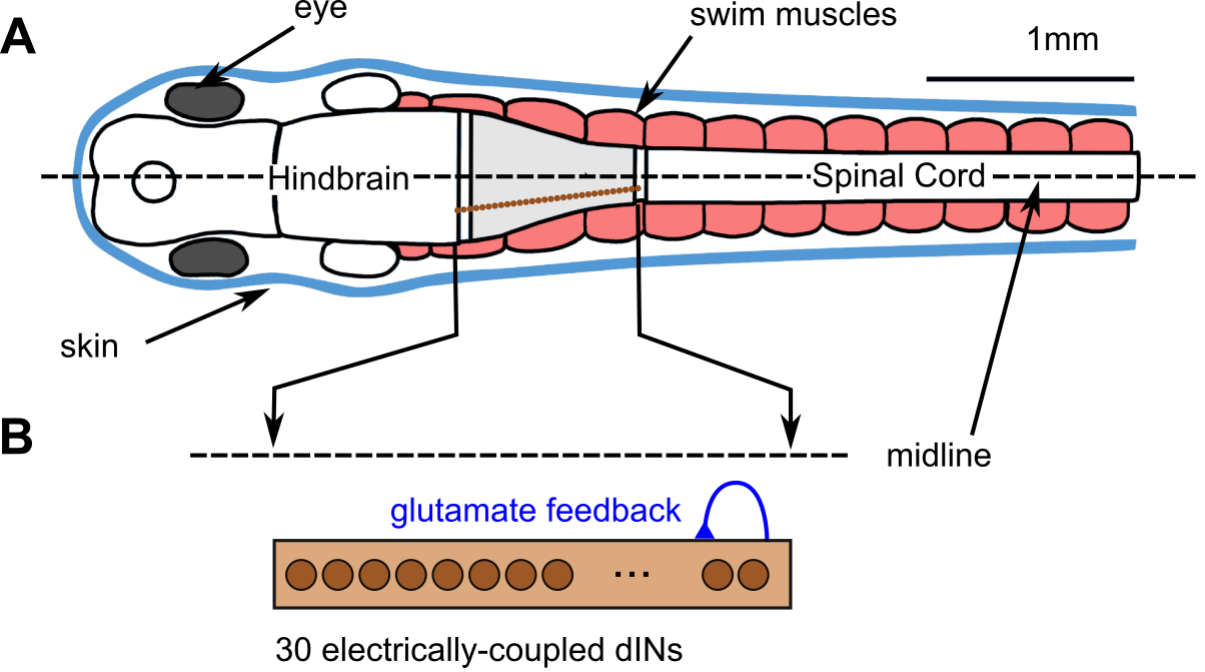
The hatchling tadpole CNS with a population of electrically-coupled dIN neurons. (A) Top view diagram of tadpole showing skin (blue), swimming muscles (pink), and CNS with hindbrain and spinal cord. The CNS region able to generate swimming rhythm when isolated (grey) contains a population of ∼30 dINs (brown) on each side. (B) On each side of the nervous system, the electrically-coupled population of ~30 dINs in the isolated region make excitatory feedback NMDAR synapses onto each other.

Our model organism, the hatchling *Xenopus* tadpole, responds to brief touch stimulation with several seconds of swimming which stops when it contacts a solid object. The sensory pathways controlling swimming and the specific populations of CNS neurons involved in generating the swimming rhythm have been defined in detail anatomically and physiologically [14,15]. During swimming, neurons fire once per cycle in antiphase with those on the opposite side and drive the firing of motor neurons. This produces alternating bends at frequencies from 10 to 25 Hz [16]. A population of reticulospinal hindbrain neurons (descending INterneurons—dINs) play a critical role. They fire first on each side on each cycle and provide synchronous, glutamatergic excitation to both their own population and to other neurons on the same side [17, 18, 19]. Mutual excitation within the dIN population underlies the self-sustained, pacemaker-like rhythmic firing which drives swimming [19]. Lesion studies have shown, firstly, that neurons in a short, 0.3 to 0.4 mm region of the hindbrain and spinal cord (grey in Fig 1A and 1B; [17]) are sufficient to generate a basic swimming rhythm and, secondly, that a single side of the CNS can generate sustained rhythm in a slightly higher frequency range (15 to 30 Hz; [20]).

Using tadpole swimming as the specific case study, we build a computational model to try to understand how small populations of electrically-coupled neurons act as pacemakers to generate a self-sustaining rhythm of firing which can be turned on and off by brief external synaptic input from experimentally defined neuronal pathways. We use a biologically based model of the minimal population of 30 electrically-coupled reticulospinal dIN neurons [17,19,21,22]. We ask whether: 1) mutual NMDAR-mediated excitation, including the ability of NMDAR activation to induce pacemaker firing, is a basis for sustained rhythm generation; 2) brief synaptic input can switch sustained activity on and off; and 3) rhythm generation based on mutual NMDAR-mediated synaptic connections can occur in networks of simpler, generic neurons. The results are of relevance to many other brain networks generating episodic rhythmic activity or the sustained firing required to control eye or limb position [23].

## Methods

### Neuron and synapse model details

A custom Python toolbox ('morphforge’) was used to perform simulations of small networks [24]. A dIN neuron model was built with a multicompartmental axon. This had a single compartment for the soma and dendrites since previous work [25] had shown that the short extent of dendrites in these young neurons means that they form an electrotonically compact unit with the soma. The axon had many compartments and an axo-axonal gap junction distribution scheme for a population of 30 dINs was used to produce a network of 30 electrically-coupled dIN neurons as in our previous study [21]. Briefly, the dINs were arranged in a column, with a spacing of 10 μm between adjacent somata. The dINs have descending axons, and gap junctions with 600 MΩ resistances were created between overlapping axons close to the soma of the more caudal neuron. The dINs contain leak, sodium, calcium and fast and slow potassium channels. The sodium and potassium currents are modelled as Hodgkin-Huxley-type currents, and the calcium model uses the Goldman-Hodgkin-Katz formulation [26–28]. Synaptic conductance at chemical synapses are calculated as the difference between two decaying exponential functions (A and B, for opening and closing) with time constants (*τ*_*o*_,*τ*_*c*_). 1ms after the presynaptic action potential crosses a threshold of 0mV in the soma (to introduce a synaptic delay), a step increase of 1 occurs in the values of A and B[13]. AMPAR and NMDAR synaptic conductance rise and fall times were taken from 13. The GABA mediated inhibitory synaptic potential times were fitted to current clamp data [29]. A scaling factor, (tc_max_) was included which is based on the time constants so that the maximum value of the difference was 1, allowing us to express all synaptic strengths as peak-conductances. AMPA and NMDA synapses were modelled using Eqs 1–7.

$$g_{syn}=g_{peak}\times \frac{B-A}{t_{max}}$$ (1)

$$i_{syn}=g_{syn}\times \left(V-E_{syn}\right)$$ (2)

$$tc_{max}=\frac{\tau_{o}\times \tau_{c}}{\tau_{o}-\tau_{c}}\times log\left(\frac{\tau_{c}}{\tau_{o}}\right)$$ (3)

$$t_{max}=exp\left(\frac{-tc_{max}}{\tau_{c}}\right)-exp\left(\frac{-tc_{max}}{\tau_{o}}\right)$$ (4)

$$\frac{d}{dt}A=\frac{-A}{\tau_{o}},\;\frac{d}{dt}B=\frac{-B}{\tau_{c}}$$ (5)

where g_syn_ is synaptic conductance with a peak of g_peak_ and E_syn_ is the reversal potential for the synaptic current. The voltage-dependence of the NMDAR synapse was modelled by introducing an additional voltage dependent term vdep^Mg2+^(V) which had no temporal dynamics, as given in Eqs 6 & 7.

$$i_{syn}=g_{peak}\times \frac{B-A}{tc_{max}}\times \left(V-E_{syn}\right)\times vdep^{Mg2+}\left(V\right)$$ (6)

$$vdep^{Mg2+}\left(V\right)=\frac{1}{1+\eta \times {\left[Mg^{2+}\right]}_{o}\times \text{exp}\left(-\gamma V\right)}$$ (7)

where η = 0.1 mM^−1^, γ = 0.08 mV^-1^ and [Mg^2+^] _o_ = 0.5 mM. In simulations of the network in zero extracellular Mg^2+^, the vdep^Mg2+^(V) term was set to 1. Feedback synaptic connections were made with a fixed probability of 0.2 between dINs.

In experiments to generalise conclusions from our models of tadpole neurons we used a population of 30 simpler, single-compartment Hodgkin-Huxley neurons with direct soma to soma electrical connections. These neurons had leak, sodium and potassium currents [27,30]. NMDAR and AMPAR mediated synaptic feedback connections were present between neurons in the population, with a connection probability of 0.2 between any pair of neurons. Inhibitory input to the population was modelled using synapses with a reversal potential of -70 mV and a peak-conductance of 3 nS. It was activated ten times at 7 ms intervals starting at 300 ms and 1400 ms. The gap junctions were modelled as resistors of 100 MΩ connecting the somata, and a pair of somata had a 0.2 probability of forming an electrical connection. The neurons had noise in the conductance densities of the membrane channels, as in [21,24] and the peak conductances of the synapses were normally distributed (σ^2^ = 0.1).

### Model of synaptic input to activate the swim network

**Fig 2:**
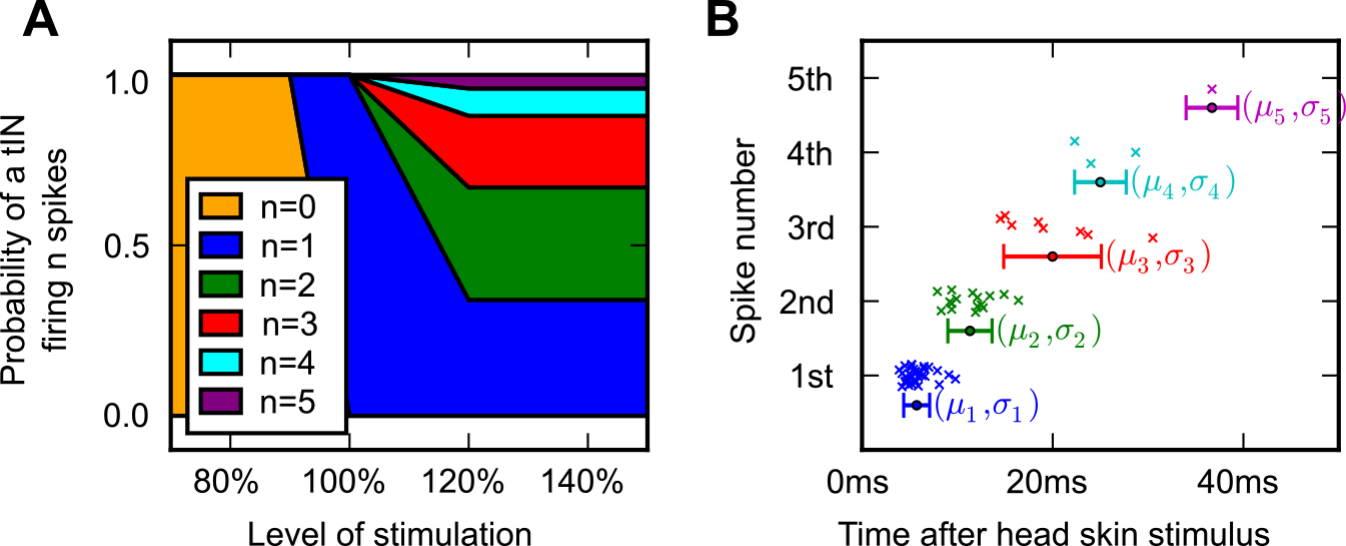
A simple generative model of spike times for a single tIN. (A) The probability distribution of a single tIN firing different numbers of spikes at levels of head-skin stimulation. (B) The times of the spikes measured experimentally are shown as coloured crosses and the means and standard deviations (μ_k_ and σ_k_) used to generate the spike times of a model tIN. Means are shown as coloured circles, standard deviations are shown as horizontal error bars.

Data from whole-cell recordings showing responses of trigeminal sensory pathway neurons (tINs) to head-skin stimuli [31] were used to model the synaptic input which reticulospinal dINs receive following head skin stimulation (see Results section for further details of this pathway). In life a tIN will fire between 0 and 5 spikes depending on the stimulus level, so a simple model was built that generated a set of spike times for a single tIN in response to graded stimuli. The stimulus strength, s, was normalised so that s = 100% corresponded to a head-skin stimulus at the threshold level required to initiate swimming. This model was used to drive EPSPs in the dINs to model sensory excitation from a biologically-realistic population of 20 tINs. In the tIN spike time model, the number of spikes fired, n, at a given stimulus level, s, was generated from the probability distribution p(N = n, S │ S = s) (Fig 2A). This simple model was based on the observations that: a) the mean threshold stimulus that leads tINs to fire a single spike is ~95% (94 ± 6%), (i.e. p(N = 1, S │ S = 95%) = 0.5); b) at 100% stimulus all tINs fire a single spike (i.e. p(N = 1, S │S = 100%) = 1.0); c) as stimulus strength increases above 100%, some tINs begin to fire multiply, but some always fire only a single spike (10/34) (p(N = 1 S│ S > 120%) = 0.3); and d) the distributions for the number of spikes firing at high stimuli (S > 120%) were estimated based on counts of spikes at higher stimulation levels [31]. Next, the number of spikes, n, fired by a model tIN was converted into timings, {t_1_, t_2_…t_n_}. The time of the k^th^ spike, t_k_ was generated from a normal distribution t_k_ = N(μ = μ_k_, σ = σ _k_), where μ_k_ and σ _k_ are the means and standard deviations of the k^th^ spike (Fig 2B). The values of μ_k_ and σ _k_ were calculated from experimental data taken at all levels of stimulation. The modelling assumed that connections were monosynaptic with 100% tIN-dIN connectivity and that a spike in a tIN had a 50% chance of causing an EPSP in each dIN [31]. A set of spike timings was constructed for the population of 20 tINs and a simple model of unreliable synaptic transmission was implemented in NEURON [32].

## Results

In all tests we used the same basic model of a linear population of 30 reticulospinal dINs on one side of the CNS and arranged in a longitudinal column (Fig 1B; [21]). All chemical synapses with defined channel opening and closing times and reversal potentials were made onto the single soma/dendrite compartment. The neurons were coupled via gap junctions between their multicompartment descending axons. In experimental work, a curious property has been observed in the dINs. They reliably fire a single action potential in response to *in situ* step current injections but they fire repetitively at low frequencies (once per cycle) during swimming. Modelling the dIN population has already shown how the single spiking behaviour could be a result of their electrical coupling, rather than their individual membrane properties [21]. The model dINs here have the same ion channels used in this previous study, including voltage-gated sodium, calcium and fast and slow potassium channels as well as passive leak channels. Since the dINs are electrically coupled, voltage clamp experiments are difficult so the models of the active channels are based on voltage-clamp recordings of other spinal neurons.

### Pacemaker responses of the dIN population to NMDA perfusion

**Fig 3:**
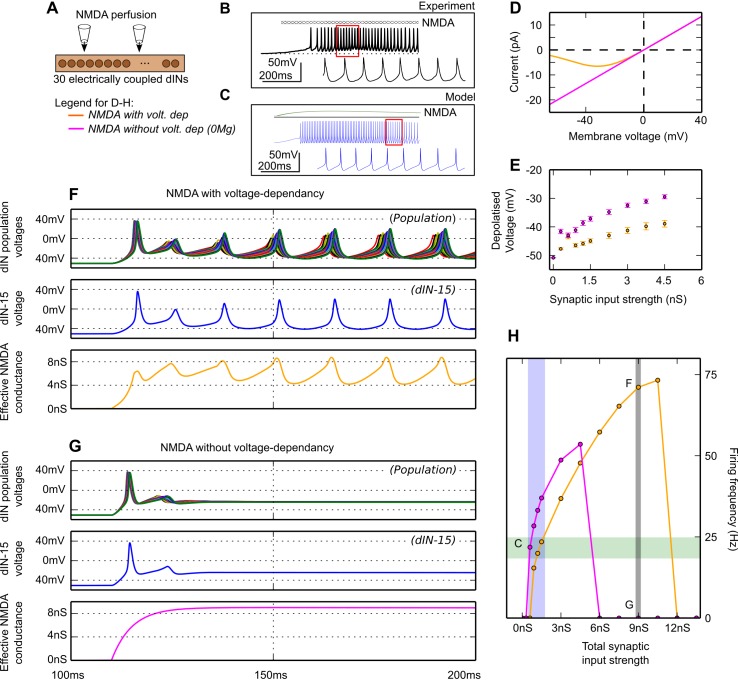
Perfusing NMDA onto the dIN population model. (A) The population of electrically-coupled dINs, onto which NMDA was perfused. (B) In life, perfusion of NMDA onto a dIN causes depolarisation and repetitive firing (black traces from Fig 2 in Li et al., 2010 [19]) where hatched bar denotes NMDA perfusion. Red box shows region expanded below. (C) Similar firing is seen in a model dIN (blue trace) where green line shows the NMDA activation reaching a maximum conductance of 1 nS. (D) Current-voltage curve of a single model dIN NMDA synapse, with (yellow) and without (pink) voltage dependence. (E) The steady state membrane potentials of dINs as a function of NMDAR conductance (with sodium channel conductance set to zero to prevent firing) with and without voltage dependency of the NMDAR as in D. (F—H) The response of the network of 30 dINs to NMDA perfusion with (F) and without (G) voltage dependency. Top: somatic membrane voltages (all dINs overlapped), middle: somatic membrane voltage of dIN number 15, bottom: conductance of NMDAR synaptic channels. (H) Plots of dIN firing frequency vs NMDAR conductance with (yellow) and without (pink) voltage dependence. Grey bar shows synaptic strength used in plots F and G (7.5nS). Blue bar shows estimated total synaptic conductance to a neuron during swimming, based on voltage clamp recordings (0.6–1.5nS). Green area shows the range of swimming frequencies observed in the tadpole.

During tadpole swimming dINs fire once on each cycle and release glutamate to excite each other [17]. The glutamate activates NMDARs and summation is proposed to produce a sustained background depolarisation. The effects of NMDAR activation have previously been examined experimentally by perfusing NMDA over one half of the tadpole CNS while recording from a dIN [19]. It was found that NMDA perfusion led to depolarisation and, if this was sufficient or combined with positive current injection, rhythmic, pacemaker-like firing at frequencies of 5–30 Hz were seen in the recorded dINs so long as Mg^2+^ was present (Fig 3B). The Mg^2+^ confers a voltage dependence on the NMDAR-mediated current [33] which is not seen during other synaptic currents or simple current injection and is not seen when extracellular Mg^2+^ is removed. During swimming, inward currents underlying the sustained depolarisation produced by summation of NMDAR excitation in the tadpole have been measured in dINs under voltage clamp and significantly correlate with swim frequency [34]. These currents (measured with Mg^2+^ present and with dINs clamped at a holding potential of -55 mV) correspond to conductances of 0.6–1.5 nS for a range of swimming frequencies of 15–20Hz.

To model rhythm generation induced experimentally by NMDA perfusion over the dIN population on one side of the tadpole CNS, we implemented a model (Fig 3A) where each dIN had a single synapse at the soma/dendrite whose NMDAR channels [13] had slow opening (5 ms) and very slow closing (10 s) time-courses. The NMDAR conductance could be simple (without extracellular Mg^2+^; modelled by setting *vdep* ^*Mg2+*^ *(V)* to 1) or have voltage dependency (with extracellular Mg^2+^; Fig 3D). To investigate the underlying effects of NMDAR activation on dINs, their firing was turned off by removing their sodium channels. Modelling NMDA perfusion by increasing NMDAR activation in all dINs together caused increasing steady-state depolarisation in each neuron (Fig 3E, yellow symbols) which was larger without NMDAR voltage-dependence (Fig 3E, pink symbols).

Just as in experiments [19], modelling NMDA perfusion by an activation of the dIN NMDARs within the physiological conductance range (< 2 nS), and including NMDAR voltage dependence, could cause the whole dIN population to fire repetitively and synchronously at frequencies like those observed during tadpole half-CNS swimming (15–30 Hz; [20]) (Fig 3A and 3C and 3H). Interestingly, robust rhythmic firing continued to increase with NMDAR activation (= synaptic input strength) for a wide range of conductances beyond the physiological level, eventually reaching frequencies much higher than observed in the tadpole (Fig 3F and 3H). Unlike the experimental findings, sustained firing also occurred in simulations without NMDAR voltage dependence (Fig 3H). Here, a lower level of NMDAR activation was required for dINs to reach firing threshold, since the resulting conductance at each synapse was higher. As synaptic input strength was increased above firing threshold, the dIN population firing frequency again increased (Fig 2H), also reaching levels higher than found experimentally in the tadpole. Firing was robust, but over a narrower range of conductances than with NMDAR voltage dependence present; at higher NMDAR conductances, dINs became unable to repolarise sufficiently to allow firing of more than one action potential (Fig 3G and 3H). We conclude that, with voltage dependence present, physiological levels of sustained NMDAR activation in the model dIN population can lead to rhythmic firing within the tadpole half-CNS swimming frequency range, and that this rhythm is sufficiently robust to continue, at higher frequencies, even at levels of synaptic input strength beyond the physiological range.

### Effects of mutual glutamate excitation on dIN responses to brief synaptic excitation

**Fig 4:**
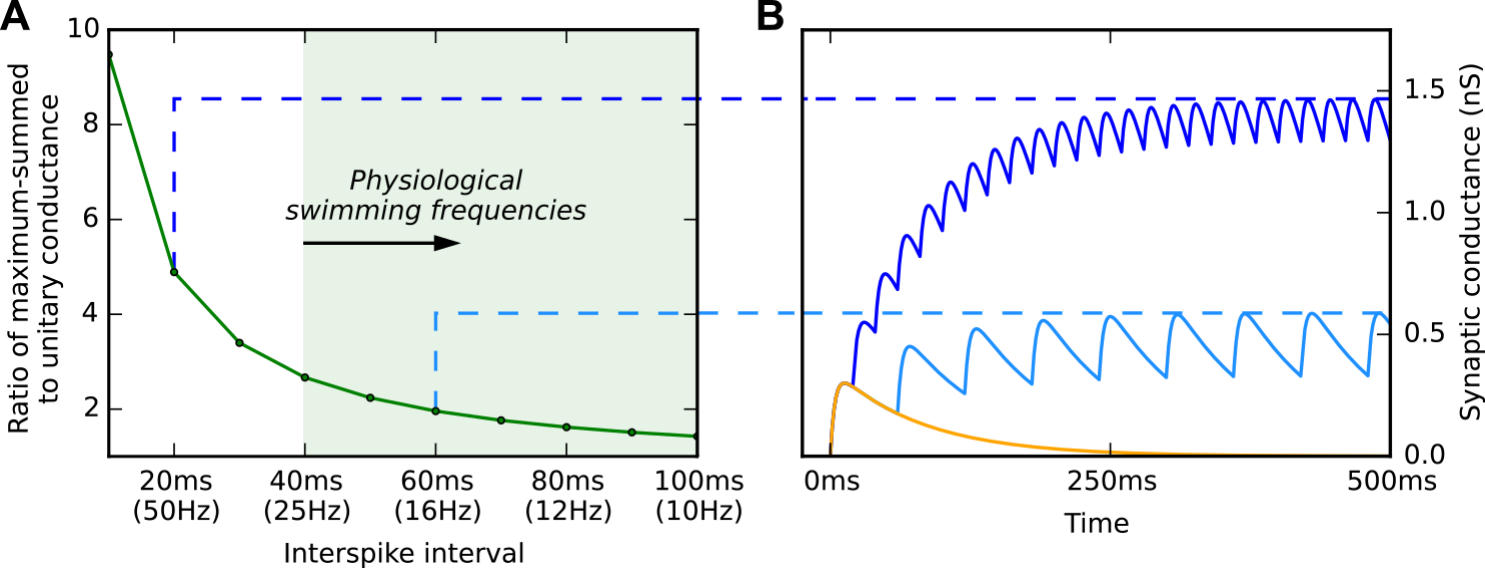
Summed conductance of feedback NMDAR synapses as a function of frequency. (A) Spike trains of different frequencies were delivered to a model NMDAR synapse with a closing time of 80 ms, and the maximum conductance recorded experimentally. (B) Faster spike trains produced a larger maximum conductance. At the frequencies of tadpole swimming, the conductances did not rise above ~3 times the conductance of a single NMDAR synaptic event (yellow trace).

A brief stimulus to the tadpole skin is normally sufficient to initiate swimming which can last for many seconds [15]. Recently, a simple pathway has been identified in the tadpole, which can initiate swimming in response to head skin stimulation [31]. Sensory neurons innervate the tadpole’s head skin, and form excitatory synapses onto a population of hindbrain trigeminal interneurons (tINs) which fire briefly to excite dINs in the hindbrain. At low levels of head skin stimulation, Excitatory Postsynaptic Potentials (EPSPs) are seen in the dIN population, but as stimulus intensity increases the whole dIN population is recruited to fire and swimming starts. This raises the question: what keeps swimming going after input from the tINs declines? Recordings have shown that dINs in the hindbrain and rostral spinal cord make reciprocal, glutamatergic, excitatory synaptic connections with each other [17]. It was therefore proposed that when the dIN population fires, these mutual synapses activate the NMDARs on other members of the dIN population, and the result acts like a perfusion of NMDA to turn on their pacemaker firing as described above (see Fig 2B of [19]). The NMDAR synaptic conductances between dINs are expected to sum from cycle to cycle during swimming to maintain a level of depolarization [17,35]. We investigated the effect of summation by driving a single NMDAR synapse with spike trains of different frequencies (Fig 4) and found that, while considerable summation was possible at high frequencies, the conductances for typical swimming frequencies of 10 to 25 Hz would sum to reach a maximum of only between 2 and 3 times the peak-conductance of a single synaptic event. Summation therefore allows the mutual excitation between dINs to be sustained from cycle to cycle without becoming excessive.

**Fig 5:**
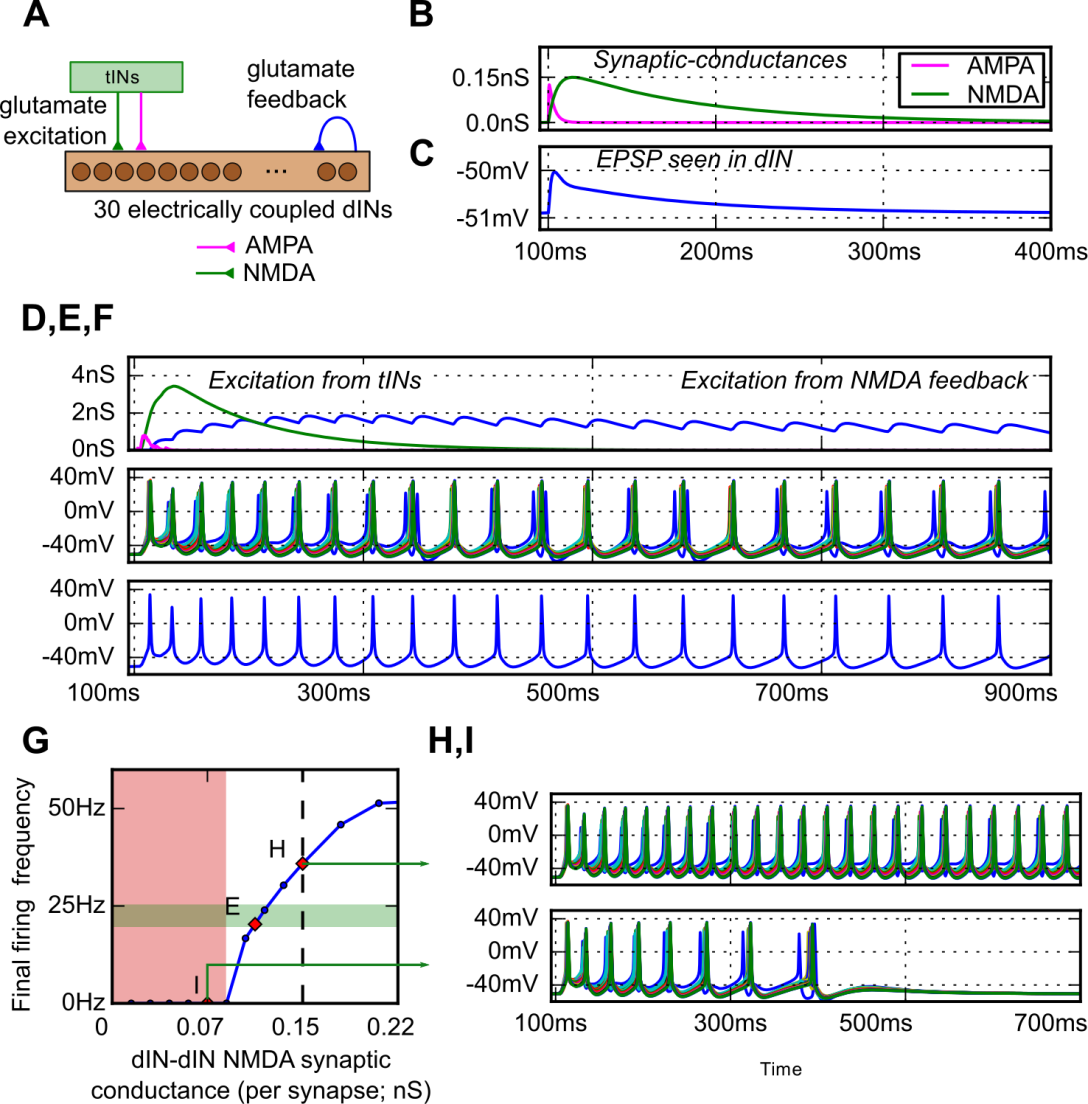
The response of a dIN network with feedback glutamate excitation to brief sensory excitation. (A) The network of 30 electrically-coupled dINs excited by sensory pathway tINs and with feedback glutamatergic synapses. (B) The conductance time-courses of the feedback dIN excitation with faster AMPAR (magenta) and slower NMDAR (green) components. (C) The resulting combined dIN to dIN EPSP. (D—F) Response to sensory input from tINs at 100 ms. (D) The conductance of sensory synapses onto one dIN (magenta: AMPAR, green: NMDAR) and summed NMDAR conductance from dIN feedback (blue). (E) After firing once to sensory input from the tINs, the dIN network shows rhythmic activity within the tadpole frequency range driven by feedback NMDAR excitation (0.11nS/synapse). (F) A single dIN voltage trace from E. (G) The effect of dIN to dIN NMDA feedback conductance on dIN firing frequency (conductance values are for a single dIN to dIN synapse). Rhythmic firing at physiologically observed frequencies is observed (green area). At low levels of synaptic strength, swimming is not reliable (red area, I). (H) At higher levels of feedback synaptic strength (0.15 nS/synapse) firing is outside normal tadpole range. (I) At lower feedback strengths (0.07nS/synapse) rhythmic firing cannot self-sustain and activity ceases after a few cycles.

We then asked if the glutamate mediated feedback excitation between members of the dIN population would allow the network to switch from rest to sustained swimming following brief synaptic excitation from a ‘sensory’ pathway? To model the sensory activation of the dIN network we introduced excitatory glutamatergic synapses mimicking those from trigeminal interneurons (tINs) onto dINs (Fig 5A). The timing and synaptic strengths of this excitation were based on experimental measures of tIN firing times in response to head skin stimulation, and EPSP amplitudes measured in dINs when tINs fired ([31]; see Methods). This synaptic input to the dINs produced long-lasting conductance increases, resulting in long-duration EPSPs (Fig 5B and 5C). If these were large enough, then the whole dIN population was recruited and fired action potentials that were synchronised by their electrical coupling [21].

To investigate whether synchronised dIN initial firing could lead to continued rhythmic firing, the electrically-coupled network of 30 dINs was used, as before. Mutual glutamatergic synaptic connections were added, with a connection probability of 0.2 between pairs of dINs (Fig 5A; glutamate feedback). We modelled these glutamate synapses with a fast AMPA component and a slow, voltage-dependent NMDA component [13,19]). The resulting EPSPs decayed in 300 to 400 ms (Fig 5C) and so could sum when dINs fired even at low rates (see above; Fig 5D blue trace). If their mutual synapses were made sufficiently strong the dINs could maintain their own rhythmic firing after brief sensory excitation, producing a switch into sustained rhythm (Fig 5E and 5F).

We explored the relationship between the strength of mutual "feedback" excitation and dIN rhythmic firing in more detail (summarised in Fig 5G). At the lowest levels of NMDAR feedback excitation above threshold, rhythmic firing of dINs was produced but was not sustained. At levels of NMDAR conductance just above this, rhythmic firing in the dIN population was reliably maintained, at frequencies like those observed in the tadpole half-CNS (Fig 5E and 5H; points indicated in Fig 5G). As with tadpole swimming, the frequency of dIN network firing increased with the strength of feedback excitation. Frequency continued to increase at conductance levels beyond the physiological range with firing frequencies eventually reaching a plateau at ~60 Hz. At these high feedback excitation levels (unitary conductances > 0.25 nS), the rhythmic activity generated was abnormal with some dINs failing to fire full action potentials. When the electrical coupling between the dINs was removed, synaptic activation of the network still led to firing that was sustained by the feedback excitation but now each dIN fired independently so there was no synchronous rhythm. In this case, the fast, chemical, synaptic excitation mediated by AMPARs was not sufficient to synchronise dIN firing.

### Synaptic termination of rhythmic activity

Having explored the switch to generation of sustained rhythmic activity in a small electrically-coupled network, we then investigated termination of this activity. In life, a swimming tadpole stops when it swims into a solid object causing pressure to the front of the head and cement gland [36]. In physiological experiments swimming can be stopped by pressure to the head skin via an identified inhibitory pathway [29]. Primary trigeminal afferent neurons innervating the cement gland and head skin form excitatory synapses onto Mid-Hindbrain Reticulospinal neurons (MHRs). These, in turn, release Gamma-Aminobutyric acid (GABA) to terminate swimming. MHRs fire multiply when the cement gland is pressed, and in response to step current injection they fire at frequencies between 40–140 Hz. It has been shown that activation of a single MHR, producing ~5 spikes, is sufficient to stop swimming in the whole tadpole [29]. This reliable sensory response provided an opportunity to use modelling to study an experimentally-defined example of rhythm termination.

**Fig 6:**
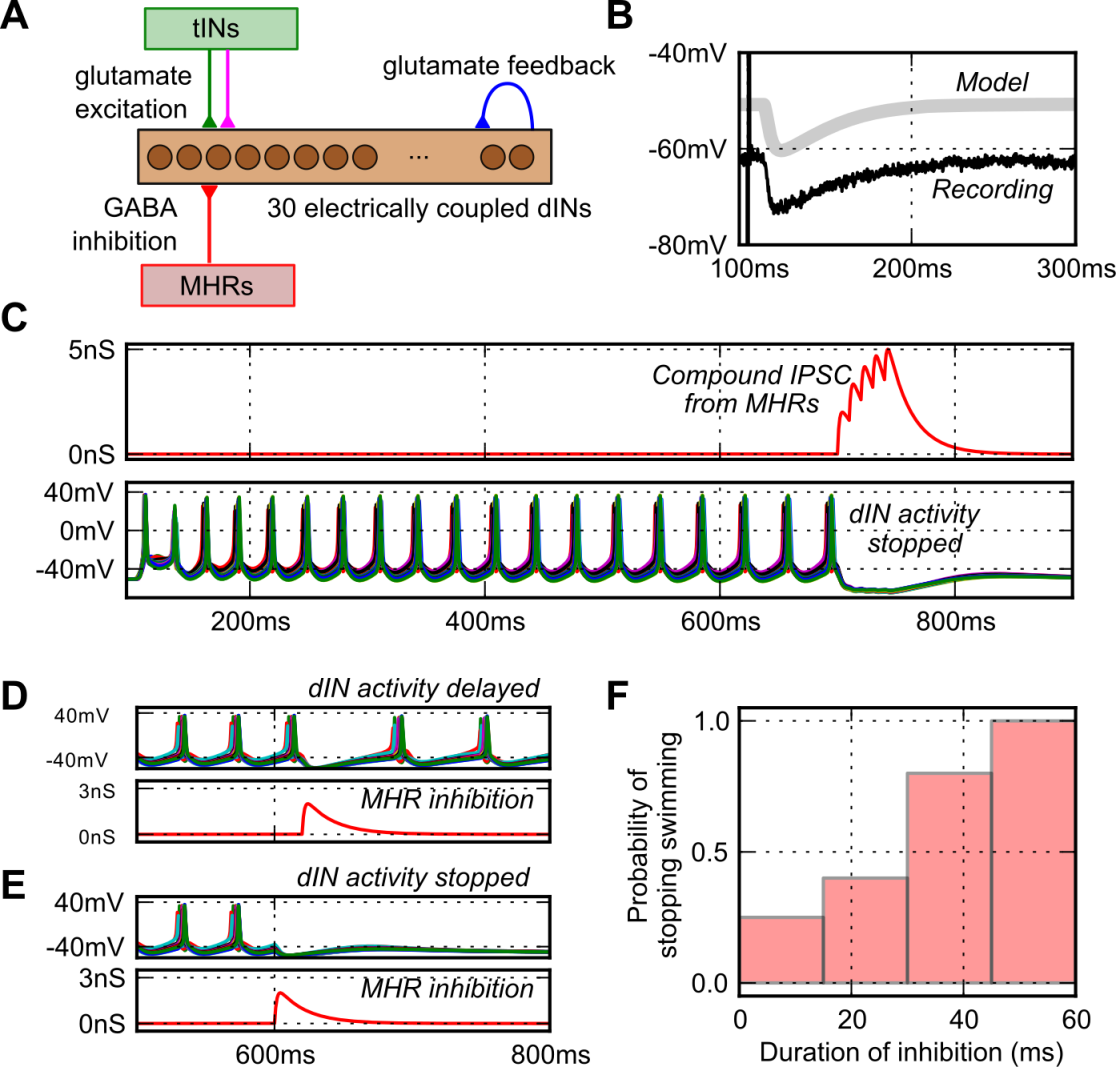
Switching off the swimming network using synaptic inhibition. (A) The dIN network with MHRs to inhibit all dINs synchronously. (B) The model GABA-A IPSP in a dIN (light grey, offset by 10 mV) matches the time-course of recorded MHR IPSP in a spinal neuron (black; Perrins et al. (2002) [29]). (C) Sensory activated rhythm generation in the dIN network (lower voltages traces), is turned off at 700 ms by five IPSPs from the MHR pathway (red conductance trace) in each dIN. (D, E). A single inhibitory synaptic event delivered to all the dINs simultaneously could delay (D) or terminate firing (E). Lower red traces show the inhibitory conductances onto a single dIN. (F) Stopping became more reliable as inhibition was made longer.

To investigate whether the dIN population could be switched off by a biologically realistic pathway, we implemented a simple model of an MHR inhibitory GABA-A synapse connected to each of the dINs (Fig 6A). The synapse specification was based on current clamp recordings of Inhibitory Postsynaptic Potentials (IPSPs) produced in tadpole spinal neurons by MHR stimulation, and from the literature (Fig 6B) (τ_o_ = 1.5 ms, τ_c_ = 20.0 ms, E_rev_ = −70 mV, g_peak_ = 2 nS; see Methods) [27,29]. The strength of the feedback glutamate/ NMDAR excitation in the 30 dIN network was set so that the swimming frequency stabilised within the swimming range at ~25 Hz. The network was activated to start producing rhythm using the tIN pathway as before and, after 700 ms, the inhibitory synapses were activated 5 times at 15 ms intervals; this is equivalent to 66 Hz which is at the low end of MHR firing frequencies [29]. This inhibitory input (Fig 6C upper trace) produced a large compound IPSP in the dINs like the MHR pathway and reliably switched off rhythm generation (Fig 6C lower traces).

We investigated the effectiveness of the stopping pathway and found that a single IPSP (maximal conductance of g_peak_ = 2 nS) delivered to all dINs simultaneously would stop spiking in 25% of simulations (Fig 6D and 6E). The effectiveness of a single IPSP depended on the time in the cycle when the inhibition arrives. It could have little effect, delay dIN firing (Fig 6D), or terminate firing (Fig 6E), and we found that IPSPs were more effective at stopping activity when they arrived later in the swim cycle. We then ran simulations in which the number of spikes (nspikes) and the interspike intervals (ISI) in the MHR were varied (nspikes:1, 2, 3, 4 & 5 spikes, ISI: 10, 15 & 20 ms, g_peak_ = 2 nS). The probability of stopping increased when the duration of inhibition [calculated as (nspikes-1) x ISI] was increased, either by adding more IPSPs or increasing the interval between them (Fig 6F). The inhibition needed to hyperpolarise the dINs for long enough to allow the background NMDA excitation to decay sufficiently to prevent further dIN firing once the inhibition has finished. In life when the tadpole’s head is pressed and swimming stops, it is likely that dINs will receive input from many MHRs, both contralateral and ipsilateral [29]. Our modelling shows that, under these circumstances, a short period of synaptic inhibition can reliably stop activity in the dIN network even when the number of inhibitory synapses from MHRs to dINs is low.

### Rhythm generation by networks of generic neurons with mutual excitation

The tadpole reticulospinal dINs that we have investigated here have a characteristic set of cellular properties [17,19,22]. To test the robustness of our findings based on the rather specific details of the tadpole reticulospinal dIN network, we asked whether less specialised populations of neurons with mutual excitatory connections also generate self-sustained activity. Positive feedback connections are often thought to lead to instability so we explored whether this was the case. In the tadpole, the dINs play a specific role in swimming and, when activated by applying NMDA, have a very low and limited firing frequency range compared to other neurons (5 to 30 Hz; [19]). We have shown that a network of neurons with dIN properties can produce stable firing at higher rates (up to ~60 Hz), but most neurons in the tadpole, and in other animals, can typically fire at even higher rates (up to 200 Hz). We therefore investigated whether populations of more typical neurons with higher intrinsic firing frequencies could generate self-sustained firing if they made mutual excitatory synaptic connections.

**Fig 7:**
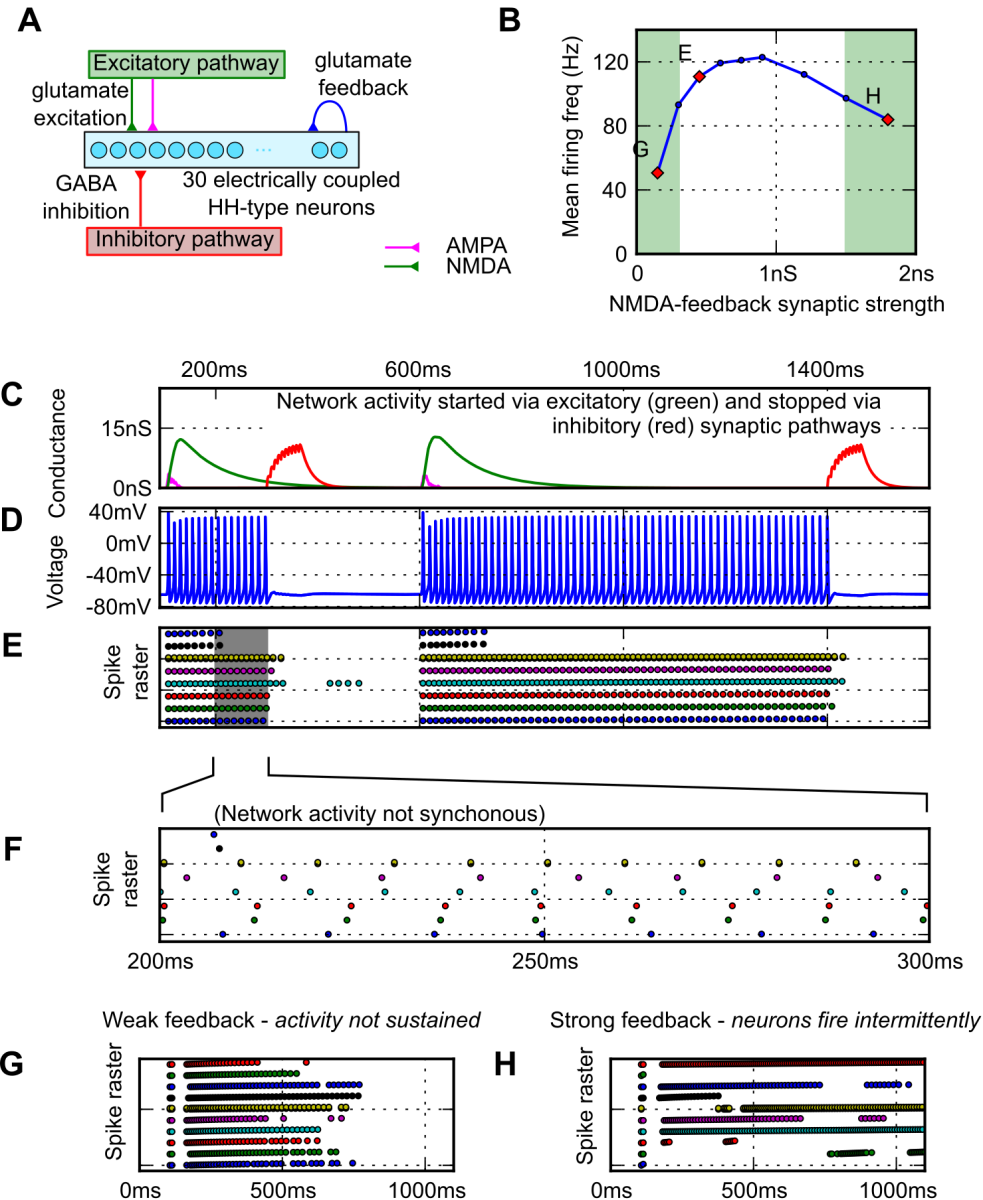
Generalising the feedback excitation activity generation mechanism. (A) The network with HH neurons replacing dINs (on/off; 100 ms/300 ms, 600 ms/1400 ms). (B) The effect of synaptic feedback strength on neuron firing (green shows regions of unstable firing). (C—F) Network activity can be switched on and off by brief synaptic input. (C) Shows the excitatory (green: NMDA, magenta: AMPA) and inhibitory (red) input to the network. (D) Voltage trace of activity in one neuron. (E—F) Shows a raster plot of action potential times for every 5th neuron in the network. (G—H) Raster plots of 10 neurons in a network with excitation at 100ms and without inhibition. (G) When feedback conductance was too low (g_peak_ = 0.15 nS); the network was unable to sustain rhythm. (left, green region of B). (G) When NMDA feedback strength was too high (g_peak_ = 2 nS), firing became unreliable.

The multicompartment dINs in the model network were replaced with single compartment model neurons with conventional Hodgkin-Huxley (HH) parameters [30]. As previously, the neurons in the network were driven by brief glutamate excitation to switch them on, had excitatory glutamate (NMDAR and AMPAR) synaptic feedback connections, were electrically coupled and had an inhibitory 'stop' input (Fig 7A; details in the Methods section). When briefly excited synaptically, the network was triggered to generate sustained firing activity provided the feedback NMDAR synaptic conductance was sufficiently large (Fig 7C–7F; indicated as E in Fig 7B). Inhibitory synaptic input (like the MHR pathway) was able to turn off this firing. As with the dIN network, when the excitatory feedback strength was low, firing activity was not always sustained (Fig 7B, left green area); members of the population generated action potentials for several hundred milliseconds but the network then returned to rest (Fig 7G; point indicated in Fig 7B). However, when excitation was increased, firing became reliable and the mean firing frequency of neurons in the network increased to a maximum of ~130Hz. As the feedback excitation was further increased, mean firing frequency decreased again and became unreliable (Fig 7B, right green area). In some cases, neurons which did not fire at initiation, would fire later once the sensory component of excitation decayed (Fig 7H top raster line). Although the model neurons were similar to each other, the random differences assigned to their synaptic and ion channel conductances led them to fire asynchronously. Low levels of electrical coupling (100 MΩ) between every pair of neurons could make small sub-groups of neurons fire together, but electrical coupling was not able to synchronise firing in the whole population. Removing electrical coupling from the network had little effect on the responses. These results show that, even without special cellular properties, small mutually excitatory populations of neurons can generate stable self-sustained firing activity which can be controlled by external synaptic input.

## Discussion

Persistent activity occurs in motor systems controlling the position of eyes and limbs and also in areas of cerebral cortex involved in short-term memory and in many sub-cortical brain regions [23]. The details of how it is generated often remain unclear but can be based on cellular or network mechanisms. Modelling a specific biologically well-defined system has allowed us to address some fundamental questions about how neuronal networks with mutual feedback excitation generate sustained activity which can be turned on and off by synaptic input. Our approach has been to build a biologically realistic computational model of a population of reticulospinal neurons in the hatchling tadpole brainstem. This model is based as far as possible on experimental data for the tadpole, and models a network with the particular properties observed in the tadpole, where there is as yet no clear evidence for either persistent currents and plateau potentials [37–40] or significant cholinergic involvement [41]. The aim was to explore the properties of this kind of organisation, and how it can be controlled (turned on and off) rather than attempting a wider survey of mechanisms for sustaining activity. Our model has provided a quantitative platform to ask questions which cannot be addressed directly *in vivo* about the mechanisms controlling action potential firing in neurons driving motor responses. To generalise our conclusions we have also studied a simple network of generic neurons with mutual synaptic excitation.

For the network controlling fictive swimming in hatchling tadpoles, we have demonstrated central roles in sustaining activity for mechanisms identified experimentally. These are: voltage-gated membrane channels; feedback glutamate excitation; and voltage-gated magnesium block of NMDA channels [17,18,42]. When these mechanisms are combined with electrical coupling [21], they are sufficient to generate self-sustaining, synchronous, pacemaker-like firing activity in a model population of reticulospinal dIN neurons in one side of the hindbrain without the need for any inhibitory feedback. We show that the voltage dependence of NMDAR channels widens the range of NMDAR channel conductances over which model neurons can be activated to fire, and that this improves the robustness of network rhythm generation. Rhythmic activity in this model population can be switched on and off robustly by brief synaptic input, and non-linearities in the cellular and synaptic dynamics avoid instabilities which might be expected in networks with positive feedback. We suggest that, although in life the dIN population forms part of a bilateral network where reciprocal inhibition sets a firing frequency of roughly twice the IPSP duration, the cellular properties of the dINs also contribute to the firing frequency [19,43]. Finally we show that networks of generic neurons with mutual NMDAR mediated excitation can sustain higher frequency, unsynchronised firing that can again be turned on and off by brief synaptic inputs. Overall, these results suggest that small synaptically-switchable networks with slow NMDAR mediated feedback excitation may be useful building blocks in the CNS toolbox and could be applicable where sustained neuron firing or rhythmic activity needs to be generated and controlled. Examples are posture, eye position and intermittent rhythmic movements, like swimming. In each case frequency could be controlled by the size of the neuron population recruited.

Cellular pacemaker mechanisms and network mechanisms involving Post Inhibitory Rebound (PIR) firing have been proposed to complement each other in producing rhythms in many networks, from molluscs to mammals, [44–52]. This also seems to be the case in the tadpole. The pacemaker hypothesis is supported by experimental studies proposing that Central Pattern Generator (CPG) neurons have intrinsic pacemaker properties [19,53,54]. However until now there seemed to be a conflict as the excitatory dIN neurons driving swimming only fired once to current injection during whole-cell recordings [17]. Recent modelling of an electrically-coupled population of 30 dINs confirmed reliable single-spike firing when step current is injected into a single dIN but showed that, in contrast, current injected into the whole model dIN population simultaneously results in synchronous rhythmic firing [21]. It appears that the single-spike firing seen experimentally is the result of reduced excitability arising from current flow into electrically-coupled neighbouring dINs at resting potential. This means that the intrinsic capacity of dINs to fire rhythmically as a whole population has remained concealed. Previous modelling work on the tadpole investigated network mechanisms of rhythm generation and proposed that reciprocal inhibitory synaptic connections between the two sides of the CNS play a role in rhythm generation by producing PIR firing in dINs that are already depolarised by their mutual excitation [13,54,55]. A role for PIR is favoured by recent experimental evidence in the tadpole showing that optogenetic silencing of reciprocal inhibitory neurons can stop the swimming rhythm [56]. This work emphasises the interaction of inhibition-based PIR and pacemaker mechanisms.

### Role of NMDAR voltage dependence in population pacemaker firing

One of the main conclusions of this modelling study is to confirm the hypothesis that a population of neurons with mutual glutamatergic synapses can generate self-sustaining pacemaker-like activity by activating their own NMDARs [17,19]. During pacemaker firing in the tadpole dINs and during rhythmic swimming activity that they drive, it is likely that several mechanisms limit firing on each cycle, including sodium channel inactivation and a slow-activating, persistent potassium current [28,42]. After each impulse, both of these mechanisms act to prevent further firing until neurons have been reset by the repolarisation of their membrane. As discussed by Tabak and Moore [57], the characteristic voltage-dependence of NMDAR activation allows sustained excitatory drive to be delivered to dINs, whilst still allowing them to repolarise to negative levels following each action potential. The voltage dependence of the NMDAR channels therefore cooperates with spike repolarising currents by partially closing synaptic conductance. This in turn permits fuller recovery from inactivation mechanisms and contributes to pacemaker firing. Our simulations reflect this process, suggesting that the voltage-dependency of the NMDAR is not an absolute requirement for rhythmic activity but it extends the range of NMDAR activation over which rhythm can be generated (Fig 3H). The simpler, non-voltage-dependent NMDAR drive to the dINs generates rhythm over a narrower frequency range because higher levels of activation prevent repolarisation and block sodium channel reactivation. NMDARs are activated in the soma/dendrite compartment whereas spike initiation occurs in the initial axonal compartments where active channels are more dense. This separation may contribute to preventing depolarisation block of spiking during NMDAR activation. Mechanisms based on the interactions of potassium currents and voltage-dependent NMDAR currents have also been found in other cases with much slower rhythmic firing [58–61].

The NMDAR-mediated feedback mechanism underlying rhythm generation in the tadpole CNS will work in other populations of neurons with much higher intrinsic maximum firing frequencies, for example classical Hodgkin-Huxley-type neurons. Our simple models of these networks showed that at the higher frequencies, electrical coupling had little effect and, as a result, these populations generated unsynchronised firing activity. Although non-rhythmic, such small populations with feedback excitation would be particularly suited to systems where activity needed to be switched on and off quickly by synaptic input.

### Comparison of model with biology

Using the tadpole as a case study we have produced a model, based closely on experimental results, of a specific small CNS neuron population with mutual NMDAr mediated excitation, which is capable of reproducing key experimental results. However, it is an oversimplification. For example, our model dIN network could fire rhythmically in the frequency range of tadpole swimming during physiologically realistic levels of excitation, but was also stable when firing faster at higher levels of excitation (~60 Hz). The model network could also fire rhythmically in the absence of NMDA channel voltage dependency (without external Mg) and this does not occur experimentally [19]. During NMDA perfusion, the tadpole reticulospinal dINs have a low maximum firing frequency (< 30 Hz), within the normal swimming frequency range, even when additional positive current is injected (Fig 3). It is possible that limits on dIN excitation may be set by restrictions on the numbers of synapses they receive which act as a saturation mechanism. The low and narrow dIN firing frequency band may also depend on limits set by their specific cellular properties. Better evidence is required on the membrane channels they express and the ways in which these might limit their upper firing frequencies in an electrically coupled population [26,59]. Higher frequencies of NMDA induced firing in our model could result from raised excitability as a consequence of increases in axonal Na channel density that were included to improve action potential propagation by offsetting the shunting produced by electrical coupling [21]. Additionally, action potentials recorded in dINs have a characteristically long rise time [13]. Voltage clamp records from other tadpole spinal neurons have suggested the presence of a fast, inactivating A-type potassium channel with opening and closing time constants < 3 ms [62]. Since A-type potassium channels are proposed to slow firing frequency in other systems, [26,63] we introduced them into the dIN model. Better fits to the rise time and shape of the action potential were found but tests showed that this had little effect on the frequency of repeated firing of model dINs so it was not included in the network model. To resolve why NMDAR activation can make our model dIN population fire too fast, why it does not lead to the 10Hz oscillations seen in experimental recordings with Mg present but when Na currents are blocked [19], and why it can generate rhythmic firing in the absence of Mg, further evidence on the membrane channel properties of dINs is needed based on new voltage clamp recordings [59]. However, such experiments will still not provide the missing evidence on the real properties of the very fine (<0.5 μm) unmyelinated axons of dINs, and this lack of evidence is a very general problem for all investigations of such fine axons [64–68].

Although this work has focused on electrically-coupled neurons on a single side of the CNS, the tadpole has bilateral populations of neurons which form connections with other neuron populations to produce rhythmic, antiphasic firing on each side of the body during swimming [13,15]. Mid-cycle inhibition from reciprocal inhibitory 'commissural interneurons' has been proposed to organise the alternation of the two sides [54, 55]. The duration of reciprocal inhibition is an important determinant of the cycle period by providing a delay on each cycle, either before a subsequent pacemaker spike or before PIR firing. This inhibition also acts as another repolarising mechanism for the rhythmic neurons, facilitating repetitive firing at high levels of excitation.

### Conclusions

Many real neuronal networks generate rhythmic or continuous activity controlled by synaptic input. Our model of a population of tadpole hindbrain neurons sustains rhythmic firing through positive feedback onto itself. In many domains, such positive feedback risks uncontrolled exponential growth and instability. However, neuronal circuits contain many saturating non-linearities which constrain firing activity and permit the use of positive feedback. All neurons have a limited upper firing frequency, which in the tadpole dINs is particularly low. Additionally, the saturation of the NMDARs at feedback synapses, inactivation of sodium conductances, and the activation of non-inactivating potassium conductances will help to prevent run-away depolarisation and firing even without synaptic inhibition. We have explored major features of the pacemaker mechanisms in the neuronal population driving tadpole swimming and have shown that similar mechanisms could also lead to stable firing at higher frequencies and in generic neuron populations. In both cases the voltage dependence of NMDARs can facilitate strong drive to neurons without causing them to lock up. We have built a model that reproduces many experimental findings although the specific channel currents which limit firing frequencies in tadpoles remain to be clarified experimentally. We have demonstrated that electrical coupling plays a crucial role in synchronizing pacemaker population firing so that, over lower frequency ranges, stable rhythmic activity is generated. Finally, we have demonstrated that small, biologically-realistic populations of neurons with mutual NMDAR mediated excitation are able intrinsically to sustain stable firing which can be switched on and off via conventional excitatory and inhibitory synaptic input pathways. Small neuron populations with these characteristics seem ideal for activating motoneurons to control posture or eye position and intermittent rhythmic actions like locomotion.

## Supporting Information

S1 Paper Simulations Contains the source code and instructions needed to run the simulations in this paper. (ZIP)
