# Supplementary figures and images for: Modelling Feedback Excitation, Pacemaker Properties and Sensory Switching of Electrically Coupled Brainstem Neurons Controlling Rhythmic Activity
Source: PLoS Comput Biol. 2016 Jan 29;12(1):e1004702. doi: 10.1371/journal.pcbi.1004702 (PMC4732667; doi:10.1371/journal.pcbi.1004702)

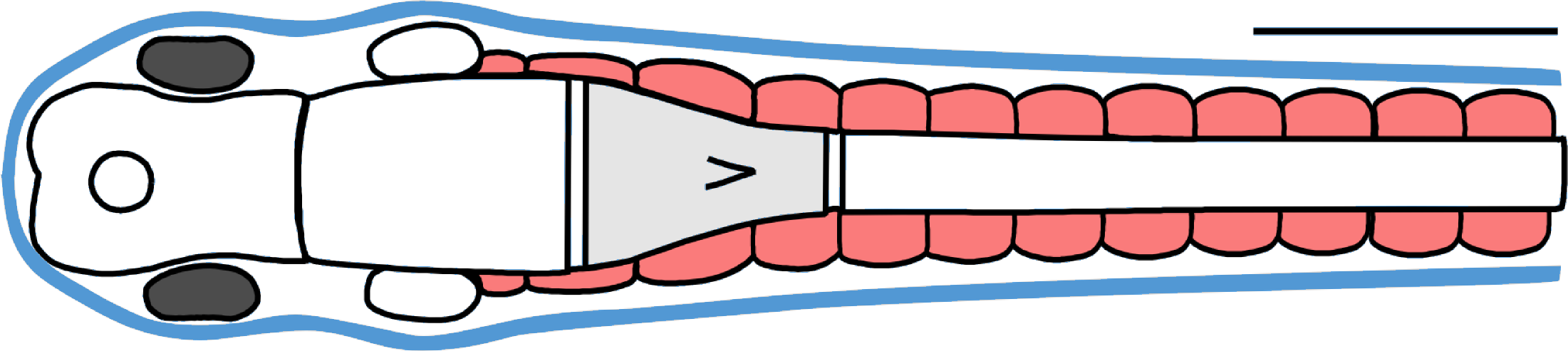

Supplement: S1 Paper Simulations — (ZIP) [file pcbi.1004702.s001.zip › paper_simulations2/paper2/src_images/tadpole_drawing.png]
